# Pre–post differences in outcomes associated with participation in the *Criar sin violencia* ^©^ brief psychotherapy protocol in an Ecuadorian sample: an observational study

**DOI:** 10.3389/fpsyt.2026.1748646

**Published:** 2026-05-15

**Authors:** Alexandra Serrano-Flores, José Ramírez-Álvarez

**Affiliations:** 1 Facultad de Salud y Bienestar, Pontificia Universidad Católica del Ecuador, Quito, Ecuador; 2 Facultad de Economía y Gestión empresarial, Pontificia Universidad Católica del Ecuador, Quito, Ecuador; 3 Facultad de Ciencias, Escuela Politécnica Nacional, Quito, Ecuador

**Keywords:** analytical psychotherapy, brief psychotheraphy, co-living violence, corporal punishment, violent discipline

## Abstract

Domestic violence against children is a form of co-living violence that is widespread worldwide. In Ecuador, verbal and physical aggression against children is often used to correct misbehavior. Exposure to this violence during childhood is associated with adverse mental health outcomes, the intergenerational transmission of domestic violence, and a an increased likelihood of involvement in criminal activity in adulthood. Individual interventions have been found to be effective to address some of the factors that endorse abusive parenting. In 2023, the protocol of brief psychodynamic psychotherapy *Criar sin violencia ^©^* was developed for this purpose. This study aimed to assess pre–post changes following the application of the Criar sin violencia ^©^ protocol, using a single-group design without a control group, to generate empirical evidence regarding its potential association with observed changes. A non-probabilistic sample (n = 38) was used. Participants were parents who used violent discipline against their children living in Quito- Ecuador. Data were collected through two questionnaires: Dimensions of Discipline Inventory (DDI) form P and SCL-90-R. The questionnaires were administered before and at the end of the application of the psychotherapeutic protocol. The Wilcoxon signed-rank test was prespecified as the primary inferential method because it provides a distribution-free assessment of within-subject change and is appropriate for studies with relatively small sample sizes. The main results were that after the application of the protocol, there was a significant reduction in psychological distress, specifically in the dimension hostility; as well as a significant reduction in the approval of the use of corporal punishment and the frequency of use of corporal punishment and psychological aggression to correct misbehavior in children. These results suggest changes in specific behaviors of violent discipline and cognitive appraisal that increase the predisposition of parents to use violent discipline against their children, that may be associated with the application of the protocol. However, limitations in sample size and measurement strategies do not allow us to establish a causal relationship. More research is necessary to strengthen the evidence about the changes related to the application of this protocol.

## 1 Introduction

Domestic violence against children is a form of co-living violence that is widespread worldwide and is often disguised as “harsh” discipline (1). The most common practices described in the literature are spanking, smacking, hitting with objects (e.g., cables, sticks, ropes), slapping, forced cold baths, pinching, ridicule, emotional manipulation, insults, and threats or coercion, which are commonly used by parents for disciplinary purposes (2–4).

Exposure to domestic violence during childhood, even when used for disciplinary purposes, has been shown to have negative short and long-term effects, including developmental delays (both physical and mental) (5–7), problems with self-regulation and externalizing behaviors in childhood and adolescence (8, 9), the intergenerational transmission of domestic violence as a perpetrator or victim (10, 11), and an increased likelihood of involvement in criminal activity in adulthood (12–14).

Despite this evidence, parents who use domestic violence against children for disciplinary purposes often hold beliefs about its harmlessness and effectiveness (1, 15, 16) largely due to the idealization of their own experiences of parental abuse during childhood which functions as a form of defensive trauma processing (3, 17) reinforced by the positive social acceptance of this practice and the absence of explicit laws banning it (18, 19).

In particular, parents who have experienced early relational trauma are at increased risk of engaging in abusive parenting especially in high-risk contexts (20, 21). Early relational trauma results from chronic exposure to abuse or neglect by parents or primary caregivers during infancy and childhood. It occurs in contexts where parents are unable to adequately respond to children’s physical and emotional needs due to the presence of intimate partner violence, sexual abuse, violent parenting, and certain psychological disorders or addictions among caregivers. This results in a violent, chaotic, and unpredictable relational environment in which caregivers are perceived as both protectors and threats, thereby generating chronic and severe stress in children (22). Early relational trauma is associated with internalizing and externalizing behaviors in adulthood (10, 23). Therefore, addressing early relational trauma in parents may be key to modifying violent discipline practices within households.

Analytical psychotherapy is a form of psychodynamic psychotherapy based on the theoretical postulates of C.G. Jung, which proposes that symbolic work through images (understood as a broad range of visual, auditory, and kinesthetic productions) activates the collective unconscious’s potential to compensate and repair maladaptive aspects of the adult personality and disruptions in psychological development (24). This psychotherapy has been found to be effective in treating a range of mental health problems in adults because it promotes the processing of complex emotions and facilitates personality restructuring, leading to stable long-term results (25–30). In the context of early relational trauma, the active and focused exploration of childhood traumatic experiences has been found to be more effective than symptom-focused treatment approaches in adults because it allows them to understand the relationship between these experiences and current psychopathological manifestations and interpersonal conflicts with enduring effects (31).

Although dynamic psychotherapies have traditionally been conceived as long-term treatments, brief dynamic psychotherapy protocols have been shown to be effective and cost-efficient alternatives for the treatment of various disorders, such as depression, anxiety, personality, eating, and somatic disorders, with outcomes comparable to those of long-term approaches (26, 32, 33) and producing significant and enduring improvements in personality structure, symptom reduction, interpersonal functioning, and daily behavior (25, 29). However, neither long-term nor short-term analytical psychotherapy protocols have been specifically developed to address abusive parenting and improve parenting skills in adults.

In regard to improving parenting, individualized interventions with parents can be effective because they focus on activating and strengthening internal mechanisms of change (20, 34, 35) while considering other factors identified in the literature, such as: tailoring the intervention to the specific needs of families (36) considering parents’ attitudes and cultural context (37) combining cognitive transformation with anger management and self-regulation strategies (38) and ensuring fidelity and precision in implementation through standardized protocols and manuals, with adequate training and supervision to therapists delivering the intervention (35, 39). However, few programs provide individual interventions for parents, most of which are based on cognitive-behavioral, attachment, and systemic approaches (20, 34, 39–41).

A growing body of research indicates that the efficacy of individual interventions to improve parenting relies on factors such as: characteristics of the parent-child relationship (41), the combination of these interventions with other comprehensive support services (social services, financial support, and health care) in highly vulnerable contexts (20), adherence to treatment through the establishment of an adequate therapeutic alliance (42), and accessibility for caregivers of different economic and educational levels (43). Furthermore, several authors agree that shorter interventions (16 sessions or less) can be highly effective in changing abusive parental behavior and are more accessible to parents both in terms of cost and time constraints (21, 39, 41).

Ecuador is a country located in South America with a total area of 256,370 km² divided into four geographical regions: Coast, Highlands, Amazonia, and the Galápagos Islands. It has 16,938,986 inhabitants, of whom 25.5% are children under 14 years of age (44). Most of the population self-identifies as mestizo and speaks Spanish as its first language, although Ecuador is a plurinational and multicultural country where indigenous people, Afro-descendants, mestizos, and migrants of various nationalities coexist (44). Its capital, Quito, is located in the Highlands and has a population of 2,679,722 inhabitants across both urban and rural areas.

Ecuador’s economic structure reflects the typical characteristics of a developing economy that is characterized by persistent challenges related to poverty, inequality, informality, and productivity. The country’s gross domestic product (GDP) is approximately USD 124 billion in 2024 with a GDP per capita of approximately USD 6,939 (45). Its economy remains highly dependent on primary sectors, particularly oil, agriculture, and mining, while the industrial and technological sectors contribute modestly to GDP. According to INEC (46), 28% of the Ecuadorian population lives in income poverty and 12.7% in extreme poverty, with rural areas being disproportionately affected. Similarly, the Gini coefficient stood at 0.47 in 2023, reflecting persistent income distribution disparities. Furthermore labor market indicators reveal that approximately 52.4% of the employed population in 2023 works in informal conditions, without access to social security or stable contracts (47). Moreover, labor productivity remains relatively low, with the average output per worker being less than one-third of that observed in advanced economies (48). These structural conditions may exacerbate family stress, limit access to support services, and increase the risk of adverse parenting practices, including violent discipline.

Regarding the national mental health system, in 2014 Ecuador began the transition from a hospital-centered and biomedical model to a community-centered model of care characterized by progressive deinstitutionalization, the establishment of outpatient services, and extramural preventive activities (49). According to the Ministry of Public Health, mental health services are provided by 1,129 establishments at the national level (approximately 20% of all public health establishments and 0.02% of all private health establishments) through psychotherapy, occupational therapy, psychiatry, social work, addiction care, and hospitalization services (50). At the public level, there are 4.2 psychologists per 100,000 inhabitants (50), whereas the regional average is 6.4 (51). By 2022, the waiting time for psychological care in the public health network was estimated to range from 4 days to 5 months, with an average consultation time of 30 minutes per patient (50). These data indicate that public services in Ecuador are far from sufficient to meet the population’s mental health care needs. This limited capacity underscores the need for scalable, cost-effective, and structured psychotherapeutic interventions that can be implemented within constrained public health systems.

With respect to violence against children, according to the latest National Children’s Survey, 45% of Ecuadorian children reported being physically or verbally abused by their parents to correct misbehavior (2). Although the Code on Children and Adolescents (52) broadly defines child abuse (art. 67), it does not explicitly prohibit certain practices, such as corporal punishment, within the family. Therefore, the Committee on the Rights of the Child has made several recommendations to Ecuador to explicitly ban violent disciplinary practices within the family, but Ecuador has not yet complied (3). Currently, Ecuador and Chile are the only countries in South America that have not reformed their legislation in this regard (53). This legal gap, combined with the high prevalence of violent discipline, highlights the urgent need for effective interventions aimed at reducing abusive parenting practices.

Children’s rights in Ecuador are protected through the Cantonal Boards for the Protection of Rights, which can act ex officio or at the request of a party to issue immediate administrative protection measures in cases of domestic violence against children. These measures may include psychosocial interventions, such as psychotherapy for parents/guardians and children, as well as other measures such as emergency foster care (52). In Quito, there are four cantonal boards for the protection of rights. During the first semester of 2023, these boards processed 1999 cases of domestic violence against children, of which 78% of perpetrators were parents (54). Approximately half of the cases processed by the boards are referred to psychosocial intervention, mostly psychotherapy for parents (4). In Quito, municipal services, non-governmental organizations, and university psychological clinics provide low-cost or free psychotherapy services, which offer brief psychotherapy (maximum 16 sessions) or counseling (maximum 5 sessions). None of these services use standardized specific individual psychotherapy protocols specifically designed to address abusive parenting. These services are crowded, with long waiting times and high dropout rates. Although the authorities are aware of the need for psychotherapeutic care for abusive parents, limited financial resources, restricted access to quality mental health services, saturation of public services, and a lack of specialized training among professionals limit families’ access to adequate and effective psychotherapeutic interventions (55). This issue highlights the need for structured, evidence-based, and scalable psychotherapeutic protocols specifically designed to address abusive parenting.

To address this gap in mental health services in Ecuador, the psychotherapy protocol *Criar sin violencia ^©^* (56) (translated as “Raising Without Violence”) was developed in 2023 to offer a standardized brief psychotherapeutic intervention specifically designed to promote changes in cognitive, emotional and relational patterns related to violent discipline in parenting. This protocol represents one of the first attempts to develop a structured, brief analytical psychotherapy intervention specifically targeting abusive parenting within a low- and middle-income country context.

The *Criar sin violencia* ^©^ is a focused psychotherapy protocol (57) based on three theoretical elements of Analytical psychology:

- The first component involves the reparative role of transference in processing early relational trauma. The protocol addresses early relational trauma processing through the analysis and management of transference and countertransference and the establishment of secure attachment by the therapist who is positioned as a parental figure (58). This allows participants to re-experience the emotions and thoughts associated with childhood trauma, but in an emotionally safe environment, thereby facilitating the observation of the relationship between their childhood experiences and the way they currently react to anger, frustration, or rejection and engage in interpersonal interactions, including those with their children. This process contributes to improving parents’ behavioral self-regulation skills.

- The second component involves the use of symbolic images within the therapeutic space to activate unconscious psychological potentials (59) that support the development of more adaptive maternal and paternal functions. This process facilitates the development of more balanced, sensitive, and flexible mental representations of the parental role, which has been identified as a key factor in the effectiveness of interventions with parents (21).

- The third component focuses on the development of self-observation skills through techniques such as creative writing and sandplay, which allow parents to explore their inner experiences and improve the self-regulation of their cognitions, emotions, and behaviors. The use of sandplay has been found to be effective for trauma processing (60) because its physical and symbolic components facilitates the processing of traumatic experiences through a nonverbal, play-based approach in an indirect and nonconfrontational way that may yield relatively rapid therapeutic effects (61, 62).

Additionally, the protocol includes a psychoeducational component that has been shown to be particularly effective in interventions with abusive parents (20, 40). Psychoeducation addresses issues such as discipline, misbehavior management, and family interaction and communication (39) as well as enhancing the understanding of the effects of abusive parenting on children in a way that encourages change (35). Psychoeducational materials should be selected by the therapists based on the child’s age and specific needs of the patient, so that they can be translated into tailored practices, ensuring relevance, applicability, and effectiveness in the real-life contexts.

Thus, the protocol offers a well-defined treatment structure that allows for adaptation to each patient’s needs without deviating from the therapeutic goal, which has been identified as a key factor in the effectiveness of this kind of intervention (21, 41).

This study aimed to assess pre–post changes associated with participation in the *Criar sin violencia* ^©^ brief psychotherapy protocol, using a single-group design without a control group with parents residing in Quito.3

This research was developed in partnership between the career of Clinical Psychology and the Free Legal Clinic of the Pontificia Universidad Católica del Ecuador PUCE, and the Secretariat of Social Inclusion of the Municipality of Quito, with the funding of the International Association for Analytical Psychology (IAAP).

The authors declare that the research was conducted without any commercial or financial relationships that could be understood as a potential conflict of interest.

## 2 Materials and methods

### 2.1 Data

A non-probabilistic sample was used. The inclusion criteria were as follows: men and women over the age of 18 with children between the ages of 0 and 18 residing in Quito, who reported using violent discipline against children at home. The exclusion criteria were cases involving criminal offenses against children and individuals with prior diagnoses of schizophrenia, intermittent explosive disorder, dissociative disorders, disruptive and antisocial behavior disorders, or personality disorders, as the psychotherapeutic protocol was not designed to address the specific clinical needs associated with these conditions Participants were required to provide informed consent prior to inclusion in the study.

The participants were recruited through two pathways: 24 participants were referred by the Cantonal Boards for the Protection of Rights of Quito as perpetrators of domestic violence against children, and 31 participants were volunteers who responded to an open call issued on social media. Thus, the initial sample comprised 55 participants.

However, 17 participants (30.9%) withdrew before completing the program. As a result, the final sample consisted of 38 participants who completed the 12 therapy sessions and the assessments before and after the intervention.

The characteristics of the final sample are described below:

Participants had between 1 and 3 children under the age of 18 years; 30 participants were women and 8 were men, aged between 26 and 60 years, and 95% self-identified as mestizo. The higher proportion of women in the sample may reflect gender differences in help-seeking behavior and caregiving roles, as reported in previous research (50). However, this gender imbalance does not imply any bias in the conclusions because the intervention is tailored to individual needs. **Table 1** details additional sociodemographic characteristics of the sample.

**Table 1:** Sociodemographic characteristics of the sample.

Marital status (number of cases)	
Separated	4
Divorced	4
Single	11
Common-law marriage	7
Widowed	3
Married	9
Gender (number of cases)	
Female	30
Male	8
Age average years	38.45
Educational level (number of cases)	
Elementary	5
Incomplete secondary	3
High school graduate	7
Incomplete tertiary education	5
Complete tertiary education	16
Fourth level completed	2
Type of family (number of cases)	
Single parent	12
Separated parents	4
Reconstituted two-parent	5
Nuclear two-parent family	16
Extended	1

The instruments used to collect the data are described as follows:

#### 2.1.1 The brief therapy protocol *Criar sin violencia* ^©^

It is a session-by-session psychotherapeutic guide designed to facilitate changes in parents’ beliefs and behaviors endorsing the use of violent discipline against children and to provide tools for respectful parenting through 12 individual psychotherapy sessions. This protocol is protected by the copyright of the Pontificia Universidad Católica del Ecuador; therefore, the following description is an authorized summary for publication.

The specific objectives of the protocol are: to elaborate narratives related to parents’ early relational trauma; to activate psychological resources that support the adaptive functioning of maternal and paternal roles; to develop self-observation and self-regulation skills; and to develop non-violent strategies that promote respectful parent–child interactions. To address these objectives, the protocol is structured into five phases as follows:

Phase 1. First interview (Session 1): An interview based on a standardized guide is conducted to identify the relationship between parents’ early relational experiences and their current parenting strategies. In addition, the therapeutic setting is discussed.

Phase 2. Elaboration of narratives of early relational trauma (Sessions 2-5): through therapeutic exploration of narratives related to childhood experiences, the client is guided toward the reinterpretation of early traumatic events, with the aim of developing a new subjective position as an adult prioritizing self-care. A standardized thematic guide is used during this phase.

Phase 3. Activation of psychological resources (Sessions 6-9): through specific analytical techniques (sandplay, fairy tales and creative writing) the client explores images and symbols representing positive aspects of parenting aimed at repairing maladaptive aspects of current functioning (59). The selection of techniques is individualized.

Phase 4. Psychoeducation for respectful parenting (Sessions 10-11): Based on real parenting situations, the client and the psychotherapist collaboratively develop strategies for effective and respectful parenting practices. Psychoeducational materials will also be usedin diverse formats (books, podcasts, short videos, etc.) along with behavioral modeling behavior and simulation to practice the strategies during the sessions.

Phase 5. Closure of the process (Session 12): This session lasts between 1.5 and 2 hours and is divided into two components. First, the achievements of the psychotherapy are evaluated and recommendations for future treatment are provided. Second, the application of the post-treatment measure.

#### 2.1.2 Revised symptom checklist SCL-90-R

The SCL-90-R (63) is a 90-item checklist evaluated on a five-point scale (0–4), reflecting subjective distress experienced over the last week. It is designed for individuals aged13 and 65 years. It measures nine primary dimensions of psychological distress: somatization, obsessive–compulsive symptoms, interpersonal sensitivity, depression, anxiety, hostility, phobic anxiety, paranoid ideation, and psychoticism. It also measures three global indices: global severity index, total positive symptoms, and positive symptomatic distress index. The instrument has demonstrated good reliability in Latin American samples, with internal consistency coefficients ranging from 0.66 to 0.97 (64, 65). Administration takes approximately 15 minutes. The instrument was administered to assess the changes in the psychological distress associated with a potential tendency toward the use of violence in child-rearing, as well as to determine whether participants met the inclusion criteria.

#### 2.1.3 Dimensions of discipline inventory questionnaire P Spanish version

The DDI form P (66) provides information about parents’ disciplinary behaviors, contextual factors and modes of implementing disciplinary practices, parents’ cognitive appraisal of discipline behaviors and sociodemographic risk factors. It is a reliable and valid tool for measuring the effects of interventions to change discipline styles and parenting practices (67). In this study, only the following two scales of the DDI were analyzed:

o Discipline Behaviors Used scale: this scale measures the frequency (daily, monthly and yearly) of using 26 discipline behaviors.
o Cognitive Appraisal of Discipline Behaviors scale: this scale measures the degree to which the parents approve or disapprove 26 discipline behaviors.

Both scales are divided into nine subscales which are classified in power assertive/punitive and non-punitive discipline. The subscales and their classification are shown in **Table 2**.

**Table 2:** Subscales of dimensions of discipline inventory (DDI).

Subscale	Discipline behavior measured	Type of discipline behavior
Corporal punishment	Shake, grab, spank, slap, smack, swat with or without objects like paddle, belts, etc. Wash child’s mouth out with soap, put hot sauce on their tongue.	Power assertive/punitive
Deprivation of privileges	Take away or withhold child’s allowance, toys, or other privileges. Send the child to bed without a meal Ground or restrict child´s activities outside the home	Power assertive/punitive
Diversion	Put the child in “time out” or give something else they might like to do instead of what they were doing wrong.	Non punitive
Explain/teach	Explain to the child what the rules are, show or demonstrate the right thing to do.	Non punitive
Ignore misbehavior	Deliberately not pay attention to misbehavior or let the child misbehave so that they would have to deal with the results.	Non punitive
Penalty tasks and restorative behavior	Give the child extra chores as a consequence for some misbehavior.	Power assertive/punitive
	Make the child apologize for misbehavior.	Non punitive
Psychological aggression	Shout, yell, try to make the child feel ashamed or guilty, acting cold or holding back affection. Tell the child that they are lazy, sloppy, thoughtless.	Power assertive/punitive
Reward	Praise or give money or other things to the child for finally stopping bad behavior or for behaving well. Tell the child they were doing a good job when behaving well.	Non punitive
Monitoring	Check on the child to see if they were misbehaving and tell the child that is being watched.	Non punitive

Data were collected between February 1, 2023, and August 31, 2024. The data collection procedure is described below:

- An initial interview was conducted with all participants to ensure compliance with the inclusion criteria, provide information about the study, and confirm participation through the provision of informed consent.
- The DDI Questionnaire form P and the SCL-90-R Inventory, both in Spanish, were administered online prior to psychotherapy and were completed by each participant with assistance from the psychotherapist.
- All participants received 12 individual brief psychotherapy sessions according to the protocol *Criar sin violencia* ^©^. The sessions lasted one hour and were held weekly. The psychotherapy was administered by senior clinical psychology students who completed their preprofessional internships between January and December 2023, as well as licensed clinical psychologists with 1 to 3 years of experience. All psychotherapists completed 20 hours of training on the theoretical principles and techniques of analytical psychology used in the program aspects of psychological and neurological development in childhood and its relationship to attachment; the psychosocial impact of violent discipline in child-rearing; and the application of the protocol.
- To monitor fidelity to the protocol and ensure it was applied consistently by all psychotherapists, individual clinical supervision was provided on a weekly basis. In addition, each session was recorded in each participant’s clinical file to ensure sessions were administered as specified in the protocol considering the level of personalization permitted by it.
- At the end of the 12 psychotherapy sessions, the DDI Questionnaire form P and the SCL-90-R Inventory, both in Spanish, were administered online and were completed by each participant with assistance from the psychotherapist.
- Initially, data collection was expected to be conducted only with cases referred to by the authority. However, the referral rate was lower than planned, which was beyond the control of the research team. To increase the sample size, volunteer participants were subsequently included. All changes to the recruitment strategy were revised and approved by the Ethics Committee for Research in Humans of the Pontificia Universidad Católica del Ecuador. Apart from this modification, there were no other adverse events or protocol deviations that could affect the study outcomes.
- The protocol was initiated within two weeks from the first contact with the participant. The 12 psychotherapy sessions were provided on a weekly basis. A justified absence from one session was permitted in exceptional cases, in which case the application of protocol was extended by one week to complete the 12 sessions. In cases of more than two consecutive absences, the protocol was discontinued to preserve measurement consistency. During the application of the protocol, no participant received any other psychological intervention.

Two databases were constructed from the collected information: one containing data collected prior to the application of the protocol and another containing data collected after the application of the protocol. All participants were assigned unique alphanumeric codes to ensure confidentiality and anonymity.

### 2.2 Statistical test

The changes in behavioral, cognitive and emotional indicators before and after the application of the protocol were evaluated using a single-group pre-post design. The Wilcoxon signed-rank test was prespecified as the primary inferential method because it provides a distribution-free assessment of within-subject change and is appropriate for studies with relatively small sample sizes ( n=38). The paired Student’s t-test was additionally used as a complementary parametric analysis of mean change. Bootstrap and permutation procedures were also included as sensitivity analyses to evaluate the robustness of the findings under resampling-based inference (68). The purpose of using multiple methods was to assess whether the observed pre-post changes were stable across parametric, rank-based, and resampling-based approaches. It is important to underline that statistically significant changes identified by these tests should not be interpreted as evidence of a causal treatment effect, since the study lacked a control group and both observable and unobservable factors may have influenced the outcomes of the psychotherapy sessions.

The Wilcoxon signed-rank test is a non-parametric test that ranks the absolute values of the paired differences, excluding zeros, as follows: The test statistic is defined as follows:


$$W\;=\;min(W_{+},\;W_{-})$$

where $W_{+}$ and $W_{-}$ are the sums of ranks for positive and negative differences, respectively. Under the null hypothesis that the paired differences are symmetrically distributed around zero (or equivalently, that the median difference equals zero), the distribution of the statistic W can be approximated by a normal distribution.

The paired t-test evaluates whether the mean difference between paired observations equals zero using the following statistic:


$$t=\frac{{\bar{y}}_{d}}{s_{d}/\sqrt{n}}$$

where ${\bar{y}}_{d}$ denotes the mean of the differences between pre- and post-treatment observations, and $s_{d}$ represents the standard deviation of these differences. Under the null hypothesis of no mean difference, the statistics t follows a Student’s t-distribution with n−1 degrees of freedom.

The bootstrap test is another non-parametric approach commonly used in program evaluations. It relies on resampling the observed differences to empirically approximate the sampling distribution of the test statistic. For a two-sided test, the p-value associated with the null hypothesis of no mean difference is computed as follows:


$$p=\frac{1}{B}\overset{B}{\underset{b=1}{\sum }}I(|{\bar{y}}_{d}{(}^{b})|\geq |{\bar{y}}_{d}|)$$

where ${\bar{y}}_{d}{(}^{b})$ is the mean of the resampled differences for bootstrap sample b, and B is the total number of bootstrap samples. Each bootstrap is obtained by resampling the observed differences with replacement. The null hypothesis is rejected when the p-value is below the chosen significance level.

Finally, the permutation test randomly flips the sign of each observed difference, assuming that positive and negative differences are equally likely, to evaluate whether the distribution of differences is symmetric around zero. For a two-sided test, the p-value is calculated as follows:


$$p=\frac{\sum_{b=1}^{B}I(|{\tilde{y}}_{d}{(}^{b})|\geq |{\bar{y}}_{d}|)}{B+1}$$

where ${\tilde{y}}_{d}{(}^{b})$ denotes the mean of the permuted differences for iteration b, and B is the total number of Monte Carlo permutations. Each permutation randomly and uniformly flips the sign of the observed differences. As before, the null hypothesis is rejected when the p-value is below the chosen significance level.

## 3 Results

**Figure 1:**
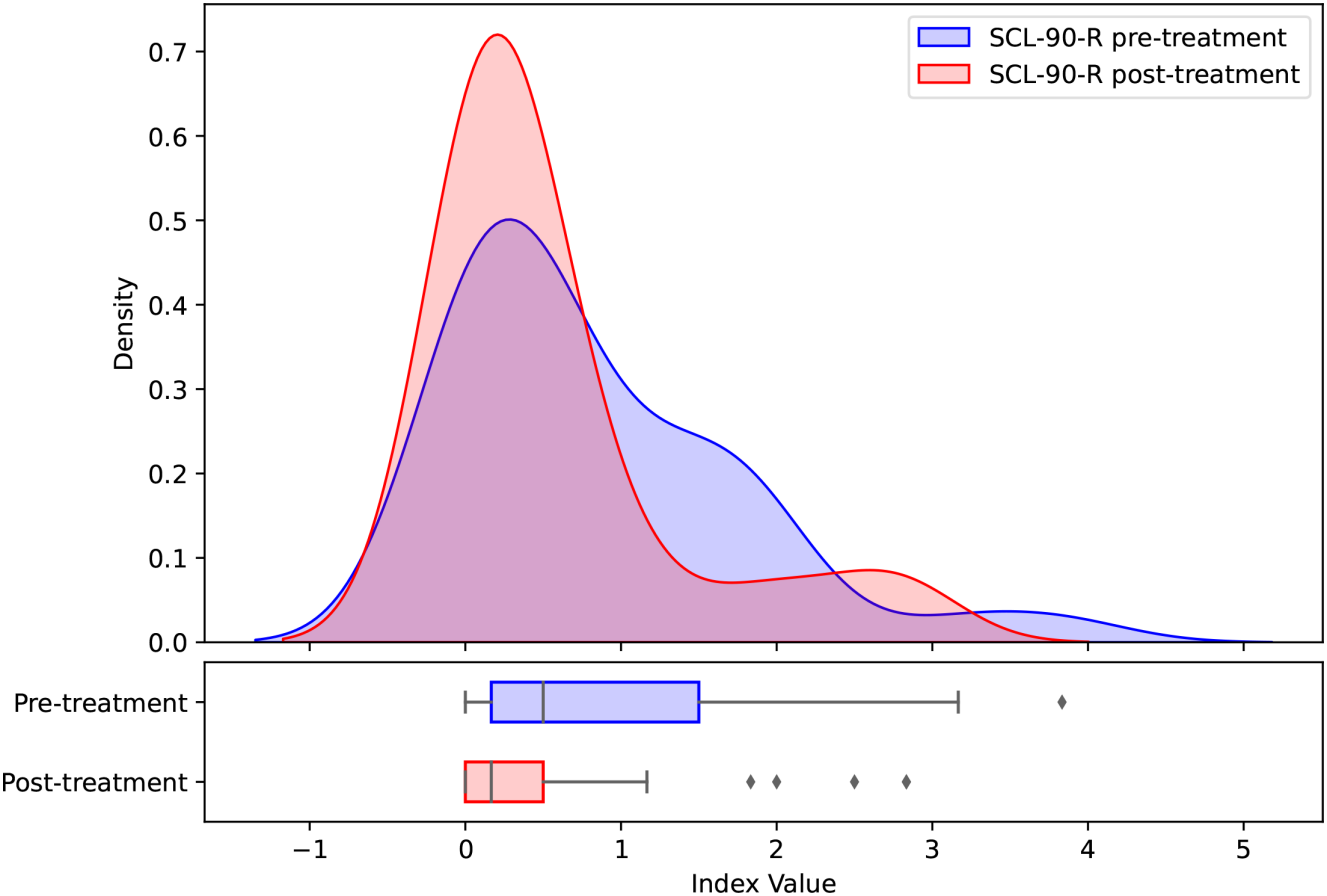
Kernel density distributions of the hostility dimension of SCL-90-R of the pre- and post-treatment samples.

The kernel density distributions of the hostility dimension of the SCL-90-R before and after application of the protocol indicate a shift toward lower values in the post-treatment distribution (**Figure 1**). Compared with the pre-treatment distribution, the post-treatment distribution appears more concentrated around lower scores and exhibits a narrower spread. In addition, the pre-treatment distribution shows greater dispersion and a more pronounced right tail, indicating the presence of individuals with relatively high baseline scores. By contrast, the post-treatment distribution is more peaked and less extended toward higher values, indicating lower scores and reduced heterogeneity in symptom levels across participants. Nevertheless, some overlap remains between the two distributions, indicating that changes were not uniform across participants.

Based on the instrument’s scoring table (59), 42% of participants scored at or above 57 in the Hostility dimension before the application of the protocol, placing them within the highest 25% of the normative distribution for aggression, anger, irritability, and related affective and behavioral manifestations. After the application, this proportion declined to 23.6%,.

According to the Wilcoxon signed-rank test, hostility scores after the application of the protocol were significantly lower than pre-treatment scores at the 5% significance level, decreasing from 0.83 to 0.56 points. This represents a reduction of 0.27 units with a small effect size (r = 0.07) and a 95% confidence interval ranging from 0.018 to 0.5405 (**Table 3**). This reduction was also supported by the other three paired-sample inferential approaches, namely the paired t-test, bootstrap test, and permutation test, all of which showed statistically significant results. The convergence of these findings across parametric, nonparametric, and resampling-based methods strengthens confidence that the observed reduction in hostility reflects a robust pre-post change that could have been associated with the psychotherapy sessions.

**Table 3:** Pre-post change in hostility scores.

Statistic	Value
Pre-treatment mean (sd)	0.83 (0.93)
Post-treatment mean (sd)	0.56 (0.8)
Mean change	-0.2703
95% CI of change	[0.018; 0.5405]
Primary test: Wilcoxon signed-rank	W =320.5, p =0.0129, r =0.0769
Sensitivity analysis: Paired t-test	T =1.9966, p =0.0267
Sensitivity analysis: Bootstrap test	p =0.0209
Sensitivity analysis: Permutation test	p =0.0256

W and T correspond to Wilcoxon statistic and t statistic, respectively. Sd, p and r correspond to standard deviation, pvalue and effect size, respectively.

Regarding the dimensions assessed by the Discipline Inventory (DDI Questionnaire Form P), the pre–post comparisons indicate a consistent shift toward lower values across both cognitive and behavioral domains associated with punitive and violent parenting practices, all statistically significant at the 1% level according to the Wilcoxon signed-rank test(**Tables 4**, **5**) and corroborated by all three sensitivity tests (**Tables 6**, **7**). In line with the pattern observed in the hostility dimension, these reductions suggest not only a decrease in central tendency but also a contraction in the intensity of corporal punishment and psychological aggression responses in the post application measurement.

**Table 4:** Pre-post change in discipline behaviors and cognitive appraisal scores related to violent discipline.

Dimensions	Mean change	Wilcoxon signed-rank
Corporal Punishment	-26.00	p =0, r =0.5385
Psychological Aggression	-107.64	p =0, r =0.5005
Corporal Punishment Approval	-0.19	p =0.0011, r =0.4341

p, p-value; r, effect size.

**Table 5:** Pre-post change in power assertive/punitive discipline behavioral and cognitive dimension scores.

Dimensions	Mean change	Wilcoxon signed-rank
Power assertive/punitive discipline	-170.35	p =0, r =0.5709
Power assertive/punitive discipline approval	-0.16	p =0.0009, r =0.14

p, p-value; r, effect size.

**Table 6:** Pre-post change in corporal scores.

Statistic	Corporal ounishment	Psychological agression	Corporal punishment approval
Pre-treatment mean (SD)	31.33 (62.9)	158.62 (236.4)	1.33 (0.4)
Post-treatment mean (SD)	5.33 (15.53)	50.97 (129.51)	1.14 (0.26)
Mean change	-26.00	-107.64	-0.19
95% CI of change	[-9.02; -46.82]	[-48.61; -178.66]	[-0.08; -0.30]
Primary test: Wilcoxon signed-rank	W =631, p =0, r =0.5385	W =614, p =0, r =0.5005	W =186, p =0.0011, r =0.4341
Sensitivity analysis: Paired t-test	T =2.669, p =0.0056	T =3.2413, p =0.0012	T =3.4973, p =0.0006
Sensitivity analysis: Bootstrap test	p =0	p =0.0001	p =0
Sensitivity analysis: Permutation test	p =0.0003	p =0.0006	p =0.0008

W and T denote the Wilcoxon and Student’s t statistics, respectively. SD, standard deviation; CI, confidence interval; p, p-value; r, effect size.

**Table 7:** Pre-post change in punitive scores.

Statistic	Punitive discipline	Power assertive/punitrive discipline approval
Pre-treatment mean (SD)	316.21 (475.46)	1.56 (0.35)
Post-treatment mean (SD)	145.85 (292.71)	1.4 (0.34)
Mean change	-170.35	-0.16
95% CI of change	[-50.20; -300.67]	[-0.07; -0.26]
Primary test: Wilcoxon signed-rank	W =645.5, p =0, r =0.5709	W =430, p =0.0009, r =0.14
Sensitivity analysis: Paired t-test	T =2.6579, p =0.0057	T =3.3653, p =0.0009
Sensitivity analysis: Bootstrap test	p =0.0023	p =0.0001
Sensitivity analysis: Permutation test	p =0.0044	p =0.0005

W and T denote the Wilcoxon and Student’s t statistics, respectively. SD, standard deviation; CI, confidence interval; p, p-value; r, effect size.

As shown in **Table 4**, substantial reductions are observed in both behavioral and cognitive components associated with violent discipline. At the behavioral level, the use of corporal punishment and psychological aggression declined with mean changes of −26 and −107.64 units, respectively, both associated with large effect sizes (r = 0.5385 and r = 0.5005). These magnitudes indicate a robust contraction in the intensity of coercive parenting practices. At the cognitive level, approval of corporal punishment also decreased significantly at the 1% level by -0.195 with a moderate effect size (r = 0.4341), suggesting a concurrent shift in attitudes that legitimize such behaviors.

Related to Power assertive/punitive discipline scores (both behavioral and cognitive) (**Table 5**) a similar pattern emerges with some heterogeneity in effect sizes between behavioral and cognitive components. The Power assertive/Punitive discipline index, which captures behavioral expressions of power-assertive and punitive strategies (corporal punishment, deprivation of privileges, penalty tasks and psychological aggression), exhibited a pronounced reduction of−170.36 units, representing one of the largest effects observed across all dimensions (r = 0.5709). In contrast, the cognitive component associated with approval of power-assertive/punitive discipline showed a moderate effect size (r = 0.14), with a statistically significant decrease of 0.164 units. This divergence suggests that while behavioral adjustments in punitive practices are substantial, cognitive change may be more gradual or resistant. Although, these scores do not exclusively represent violent discipline, DDI authors suggest power-assertive/punitive discipline practices are more likely to involve elements of expressive aggression (66).

These findings support a coherent pattern in which behavioral changes appear more pronounced than cognitive changes in the measurements after application of the protocol. Nevertheless, the concurrent reductions across domains indicate a multidimensional improvement that aligns with the objectives of the psychotherapy protocol.

## 4 Discussion

### 4.1 Reduction of psychological distress related to hostility

The comparison between pre- and post-treatment data of the SCL-90-R shows a significant reduction in the Hostility subscale, which measures aggressiveness towards others, including feelings of annoyance, resentment, rage, and potential acts of aggression (69). This subscale is useful for identifying individuals with difficulties in anger management and externalizing behaviors (70).

While this indicator does not directly quantify reductions in violent discipline, prior research suggests that parents tend to use domestic violence against children when experiencing emotional distress (16, 17). Therefore, the observed decrease in parental aggressiveness and hostility in this study may be associated with a reduction in the use of violent discipline practices. This result is consistent with previous studies indicating that parenting programs that promote emotional regulation and anger management are particularly effective in reducing violent parenting behaviors (71–73).

### 4.2 Cognitive and behavioral changes

Regarding discipline behaviors used by parents, post-treatment measures show a decrease in the frequency of use of corporal punishment and psychological aggression used to address children’s misbehavior. Both behaviors, corporal punishment and psychological aggression, are considered forms of violent discipline and can even be categorized as adverse childhood experiences (7, 10). Therefore, the observed decrease in their use constitutes a specific indicator of reduced violent discipline against children, which may be associated with the application of the protocol. These results are similar to other research that also found that the decrease of use of corporal punishment and psychological aggression is a positive outcome of parent training programs (74–76) and individual psychotherapy aimed at improving parenting practices (77, 78).

Additionally, it was observed that there was a decrease in the corporal punishment approval subscale. Although this indicator measures opinions and attitudes, the literature states that there is a strong relationship between cognitive evaluation of disciplinary methods and the likelihood of their use, especially in the context of violent discipline (8, 15, 17, 79). In some cases, cognitive evaluation also reveals parents’ primary orientation toward a specific disciplinary practices, while the observable use of such practices may vary depending on child misbehavior (66). The observed change in the opinions and beliefs that favor violent discipline in the post-treatment measurements may therefore be interpreted as a positive outcome associated with the application of the psychotherapeutic protocol, as it reflects a lower parental willingness to use violence against children. This interpretation is consistent with prior research indicating that changes in parental attitudes are linked to reductions in the acceptance and potential use of violent disciplinary practices (78, 80, 81).

### 4.3 Adherence to psychotherapy and program compliance

Adherence to psychotherapy was reflected in the retention rate of 69% of participants who completed the program. Generally, a higher adherence to the programa is associated with positive psychotherapy outcomes (82, 83). These findings are encouraging, as there is still a strong stigma attached to attending psychological therapy in Ecuador (50). According to the literature, a good retention rate could be related to the therapist’s empathetic attitude, the quality of the therapeutic alliance, the therapists’ adaptability and flexibility in tailoring their technique to the patient’s needs, and the therapists’ personality that contribute to participants feeling supported and engaged (25, 82, 83).

### 4.4 Limits of the current research and future projections

Although the findings of this study provide evidence of pre–post changes across multiple domains, several limitations should be acknowledged. First, regarding the sample size, although the observed changes may be clinically meaningful, the relatively small sample limits the generalizability of the results.

Second, regarding the measurement strategy, a pre–post measurement strategy was used with psychometric tools. However, it was not possible to include a control group in this research for the following reasons: a) an important part of the sample was composed of referrals from the child protection authority, which made it impossible to predict when a certain sample size would be reached, as referrals depended on the complaints received by the Cantonal Boards for the Protection of Rights of Quito; b) most of the participants were parents who had been reported for domestic violence against children, so in order to prioritize children´s safety, all cases were required to receive psychotherapy. Moreover, it was not possible to compare the results with another group receiving alternative psychotherapy because no treatment under similar conditions was available during the data collection period in the same geographical context. The study focuses on within-subject changes, which are less sensitive to sample size than between-group comparisons. Therefore, given limitations in sample size and measurement strategies, the findings of this research should not be interpreted as causal effects. Future research should aim to include randomized clinical trials to strengthen and extend empirical evidence. The present findings nonetheless support continued efforts to evaluate this psychotherapeutic protocol.

Regarding the existence of previous research on brief analytical psychotherapies to improve parenting, a lack of studies was identified on this topic. Owing to the lack of specific references, both the literature review and the discussion were constructed with evidence drawn partially from other research in the fields of analytical psychotherapy and parenting programs.

Finally, this study identifies two areas for improvement in the implementation of the psychotherapeutic protocol: a) combining individual interventions with group modalities enhance effectiveness, and b) linking the intervention to broader frameworks that include actions to reduce parents’ economic stress, limited family and social support networks, and intimate partner violence to ensure that participating parents have adequate conditions for respectful parenting.

### 4.5 Conclusions

Violence is a complex and systemic phenomenon rooted in domestic violence and violent parenting. Accordingly, efforts to promote social well-being should begin with building safer homes for children and families.

Domestic violence against children is highly preventable with appropriate psychosocial interventions, including psychotherapy. However, in Ecuador, these interventions are quite inaccessible and unaffordable for most families. The development and implementation of specialized and standardized brief psychotherapeutic treatments could address this gap by providing low-cost interventions with high impact.

The results of this research show changes in specific behaviors of violent discipline and in cognitive appraisal related to the predisposition of parents to use violent discipline against their children, which may be associated with the application of the psychotherapeutic protocol *Criar sin violencia* ^©^. The observed retention rate in the program could be related to the flexibility of the protocol in being tailored to individual needs. However, limitations in sample size and measurement strategies preclude establishing a causal relationship. Further research is required to strengthen the evidence regarding changes associated with the implementation of this protocol.

The results of this research suggest that analytical psychotherapy can be adapted through standardized protocols to meet the needs of public health services. This is relevant in light of the prevailing assumption that only systemic and cognitive behavioral approaches are suitable for this field (84, 85), despite existing evidence that psychodynamic therapies are effective in a broad range of mental health problems and can be offered in brief, low-cost formats.

## Data Availability

The raw data supporting the conclusions of this article will be made available by the authors, without undue reservation.
